# Accuracy and reproducibility of four T_1 _mapping sequences: a head-to-head comparison of MOLLI, ShMOLLI, SASHA, and SAPPHIRE

**DOI:** 10.1186/1532-429X-16-S1-O26

**Published:** 2014-01-16

**Authors:** Sébastien Roujol, Sebastian Weingartner, Murilo Foppa, Kelvin Chow, Keigo Kawaji, Kraig V Kissinger, Beth Goddu, Sophie Berg, Peter Kellman, Warren J Manning, Richard B Thompson, Reza Nezafat

**Affiliations:** 1 Medicine, BIDMC/Harvard Medical School, Boston, Massachusetts, USA; 2 Radiology, BIDMC/Harvard Medical School, Boston, Massachusetts, USA; 3 Computer Assisted Clinical Medicine, University Medical Center Mannheim/Heidelberg University, Mannheim, Germany; 4 Biomedical Engineering, Faculty of Medicine and Dentistry/University of Alberta, Edmonton, Alberta, Canada; 5 National Heart, Lung, and Blood Institute, National Institutes of Health, Bethesda, Maryland, USA

## Background

Quantitative myocardial T_1 _mapping provides in-vivo tissue characterization for assessment of cardiomyopathies. Pre and post-contrast T_1 _maps can be used to calculate the extracellular volume fraction (ECV) to detect diffuse myocardial fibrosis. Several imaging approaches have recently been proposed for measuring T_1 _values [1-4], but no head-to-head comparison has been reported to cross-examine their accuracy and reproducibility. In this study, we compared both T_1 _maps and ECV measurements from the following techniques: Modified Look-Locker Inversion Recovery (MOLLI) [1], Shortened MOLLI (ShMOLLI) [2], Saturation recovery single-shot acquisition (SASHA) [3], and SAturation Pulse Prepared Heart rate independent Inversion-REcovery sequence (SAPPHIRE) [4].

## Methods

The four T_1 _mapping methods were implemented on a 1.5 T Phillips scanner using a b-SSFP readout (TR/TE/α = 3.1/1.5 ms/70°, FOV = 360 × 337 mm2, voxel size = 1.9 × 2.5 mm2, slice thickness = 8 mm, SENSE factor = 2). In a phantom experiment, the four methods were each repeated 10 times and were compared to the gold standard T_1 _measurements obtained using spin echo acquisitions (15 inversion times from 100 ms to 3000 ms). In-vivo analysis experiments was performed in 8 healthy subjects (38 ± 19 y, 4 m), and in 10 patients (56 ± 14 y, 6 m). Pre-contrast imaging was performed twice with the four methods. Healthy subjects were removed from the bore between the two pre-contrast scans to simulate a separate exam. Post-contrast T_1 _mapping was performed twice at 15 and 30 mins post-injection. T_1 _maps were reconstructed offline using an in-house platform and were analyzed by a blinded observer. In all T_1 _maps, the septum and the blood pool were manually delineated, and an ECV value was then computed from each pre and post-contrast T_1 _map pair. For each method, T_1 _measurement variations between the two sets of pre-contrast images and ECV measurement variations generated from the second pre-contrast T_1 _and each of the two post-contrast T_1 _data were examined.

## Results

SASHA and SAPPHIRE were more accurate but less reproducible than MOLLI and ShMOLLI for T_1 _mapping in phantom experiments. MOLLI was more reproducible than ShMOLLI and SAPPHIRE was more reproducible than SASHA. There was a trend for MOLLI and ShMOLLI to be more reproducible than SASHA and SAPPHIRE for pre-contrast T_1 _mapping in all subjects. There was no statistical significant difference in ECV measurement reproducibility among the four methods in both healthy subjects (One-way ANOVA, p = 0.51) and patients (p = 0.35). However, MOLLI and ShMOLLI yielded large errors in the derived ECV values due to error propagation of T_1 _measurements.

## Conclusions

Both SASHA and SAPPHIRE T_1 _sequences yield excellent accuracy, but with lower reproducibility compare to MOLLI and ShMOLLI. Reproducibility of ECV measurements is similar with all methods, but MOLLI and ShMOLLI demonstrated large systematic errors.

## Funding

NIH R01EB008743-01A2

**Figure 1:**
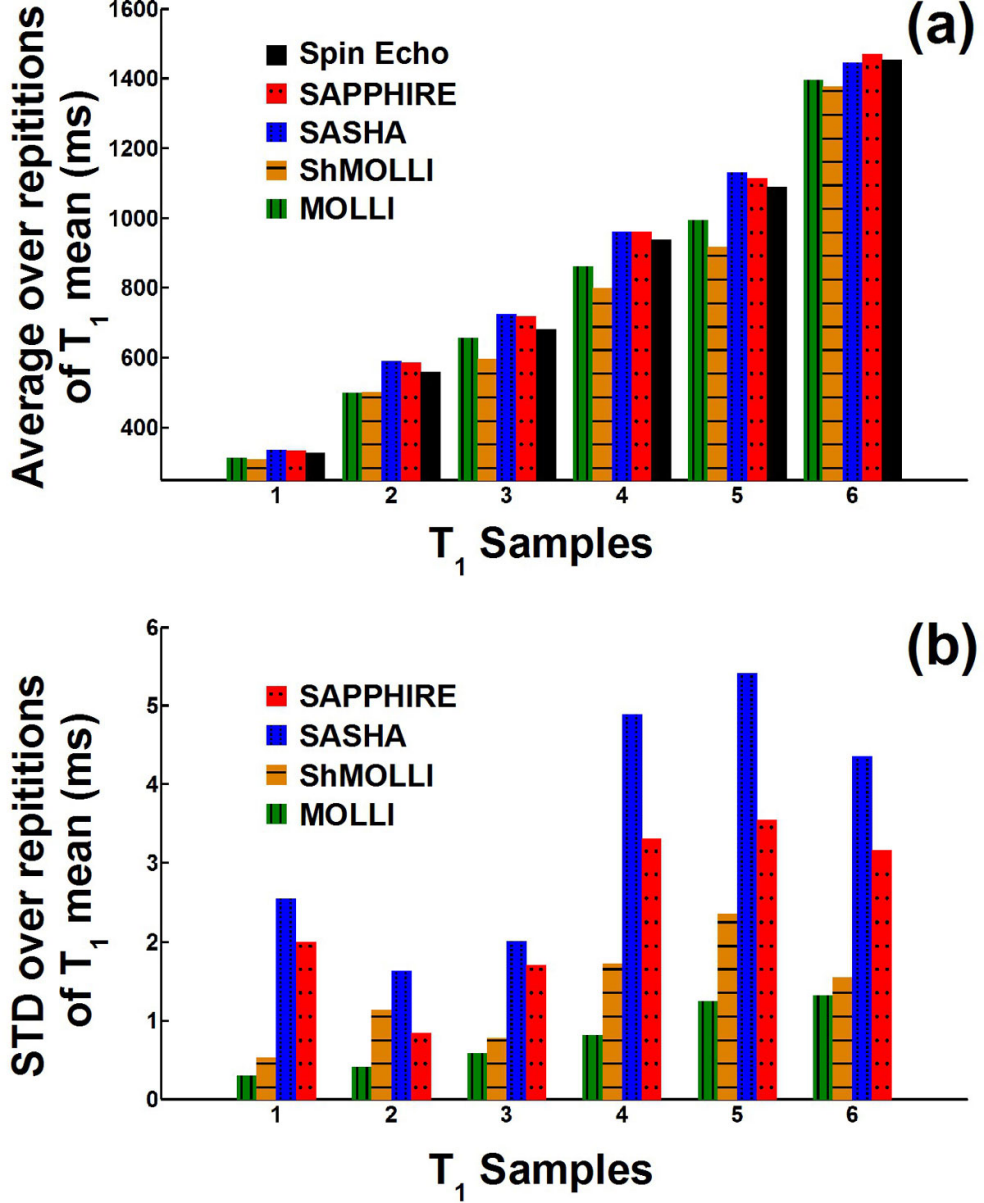
**Reproducibility of T_1 _measurements in phantom containing T_1 _samples from 300 ms to 1450 ms**. MOLLI and ShMOLLI were less accurate and more reproducible than SASHA and SAPPHIRE. SAPPHIRE was also more reproducible than SASHA while having similar accuracy.

**Figure 2:**
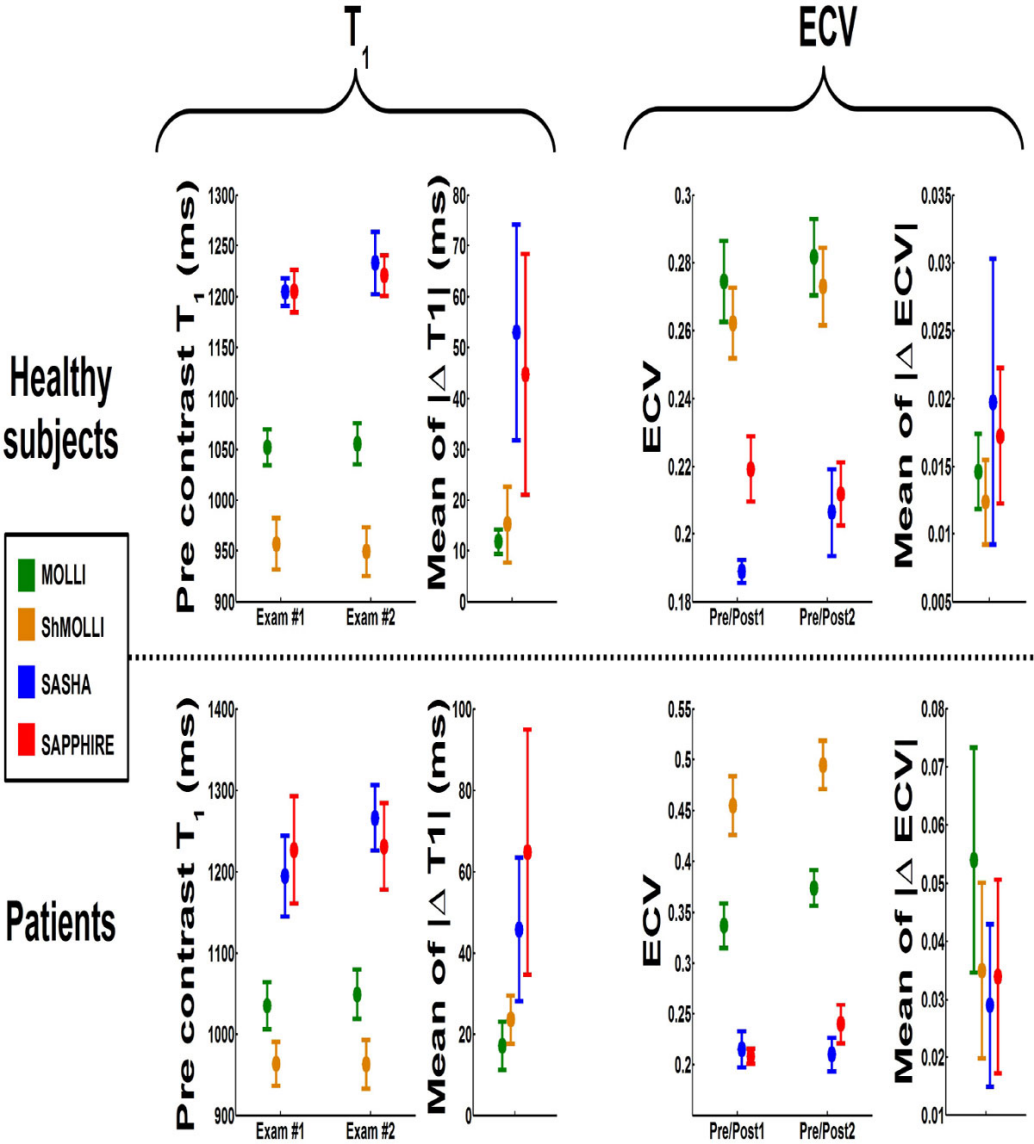
**Reproducibility of T_1 _and ECV measurements in healthy subjects and patients**. MOLLI and ShMOLLI tend to be more reproducible than SASHA and SAPPHIRE for pre-contrast T_1 _mapping. No statistical significant difference was found among the four methods in term of reproducibility of ECV measurements.
